# Effect of pre-discharge cardiopulmonary fitness on outcomes in patients with ST-elevation myocardial infarction after percutaneous coronary intervention

**DOI:** 10.1186/s12872-019-1189-x

**Published:** 2019-09-06

**Authors:** He Cai, Yang Zheng, Zhaoxi Liu, Xinying Zhang, Rongyu Li, Wangshu Shao, Lin Wang, Lin Zou, Pengyu Cao

**Affiliations:** The Cardiovascular Center, First Hospital of Jilin University, 71 Xinmin Road, Changchun, 130021 Jilin China

**Keywords:** Percutaneous coronary intervention, Cardio-pulmonary exercise, Cardiac rehabilitation, ST-segment elevation myocardial infarction

## Abstract

**Background:**

The purpose of this study was to analyze cardiopulmonary fitness in Phase I cardiac rehabilitation on the prognosis of patients with ST-Elevation Myocardial Infarction (STEMI) after percutaneous coronary intervention (PCI).

**Methods:**

The study enrolled a total of 499 STEMI patients treated with PCI between January 2015 and December 2015. Patients were assigned to individualized exercise prescriptions (IEP) group and non-individualized exercise prescriptions (NIEP) group according to whether they accept or refuse individualized exercise prescriptions. We compared the incidence of major cardiovascular events between the two groups. IEP group were further divided into two subgroups based on prognosis status, namely good prognosis (GP) group and poor prognosis (PP) group. Key cardio-pulmonary exercise testing (CPX) variables that may affect the prognosis of patients were identified through comparison of the cardio-respiratory fitness (CRF).

**Results:**

There is no significant difference in the incidence of cardio-genetic death, re-hospitalization, heart failure, stroke, or atrial fibrillation between the IEP and the NIEP group. But the incidence of total major adverse cardiac events (MACE) was significantly lower in the IEP group than in the NIEP group (*P* = 0.039). The oxygen consumption (VO_2_) at ventilation threshold (VT), minute CO_2_ ventilation (E-VCO_2_), margin of minute ventilation carbon dioxide production (△CO_2_)_,_ rest partial pressure of end-tidal carbon dioxide(R-P_ET_CO_2_), exercise partial pressure of end-tidal carbon dioxide(E-P_ET_CO_2_) and margin of partial pressure of end-tidal carbon dioxide(△P_ET_CO_2_) were significantly higher in the GP subgroup than in the PP subgroup; and the slope for minute ventilation/carbon dioxide production (V_E_/VCO_2_) was significantly lower in GP subgroup than in PP subgroup (*P* = 0.010). The VO_2_ at VT, V_E_/VCO_2_ slope, E-VCO_2_, △CO_2_, R-P_ET_CO_2_, E-P_ET_CO_2_ and margin of partial pressure of end-tidal carbon dioxide CO_2_ (△P_ET_CO_2_) were predictive of adverse events. The VO_2_ at VT was an independent risk factor for cardiovascular disease prognosis.

**Conclusions:**

Individualized exercise prescription of Phase I cardiac rehabilitation reduced the incidence of cardiovascular events in patients with STEMI after PCI. VO_2_ at VT is an independent risk factor for cardiovascular disease prognosis, and could be used as an important evaluating indicator for Phase I cardiac rehabilitation.

## Background

Acute STEMI is a leading cause of mortality and morbidity globally. STEMI leads to fatal conditions such as heart failure and sudden cardiac death, and results in an enormous psychological and financial burden on patients and the society [1]. Medication, coronary artery bypass grafting, and percutaneous coronary intervention (PCI) can reduce the morbidity and mortality in patients with STEMI [2, 3]. Medication is a basic treatment for patients before/after PCI. Compared with coronary artery bypass grafting, PCI provides an effective treatment for coronary artery stenosis. However, PCI operation may lead to coronary spasm, endothelial cell injury, and even restenosis or thrombus; moreover, a poor prognosis may still exist in patient with STEMI after PCI [4].

Cardiac rehabilitation (CR) has been found to improve the prognosis for patients with STEMI after PCI [5]. CR includes nutritional therapies, weight loss programs, management of lipid abnormalities with diet and medication, blood pressure control, diabetes management, stress management and physical exercise, and can help the recovery of physical function in patients with cardiac disease or recent cardiac surgeries. Therefore, PCI associated with CR have been recognised internationally as the preferred treatment of STEMI, now PCI combined with CR has become the internationally recognized effective treatment for patients with STEMI. Exercise training is an important part of CR, which not only improves cardiopulmonary fitness and physical activity, but also reduces the morbidity and mortality in patients with STEMI. However, as physical exercise intensifies, it also comes with certain risk. Currently, there is no specific aim for cardiopulmonary fitness in Phase I cardiac rehabilitation according to the American Heart Association (AHA) guidelines [6, 7]. The purpose of this study is to analyze cardiopulmonary fitness in Phase I cardiac rehabilitation on the prognosis of patients with STEMI after PCI, we reviewed all the information and CPX results of STEMI patients after PCI before the discharge and analyze long term prognosis of STEMI patients based on the exercise tolerance.

## Methods

### Patients

This retrospective study included a total of 586 STEMI patients treated with PCI in the Department of Cardiology at the First Hospital of Jilin University, between January 2015 and December 2015. After excluding 46 patients who were lost to follow-up and 41 patients who were not administrated with exercise prescription (Table 1), data from 499 STEMI patients were used in the final analyses. The study protocol was approved by the Institutional Review Board of each hospital.

**Table 1:** The reason of not administering exercise prescriptions for the 41 patients

	Number of patients
Herpes zoster	1
Multiple serous cavity effusion	1
Hepatic renal failure	2
Acute onset of chronic obstructive pulmonary disease	2
Cerebral infarction sequelae	8
Uremia	1
Ankylosing spondylitis	1
Diabetic ketosis	1
Diabetic foot	1
Systemic lupus erythematosus	1
Gastrointestinal ulcer	1
Cardio genic shock	1
Severe arrhythmia	8
Right intercalf venous thrombosis (acute phase)	2
Tumours	7
After aortic stent implantation	1
Second degree scald of left thigh	1
Left ventricular apical thrombosis	1
Total	41

Patients data including age, sex, cardiac function and test indices were collected, including white blood cell (WBC), hemoglobin (HGB), creatinine (Cr), glutamic pyruvic transaminase (AST), glutamic pyruvic aminotransferase (ALT), total cholesterol (TC); high-density lipoprotein cholesterol (HDL-C) and low-density lipoprotein cholesterol (LDL-C). All 499 patients had no contraindication of cardiopulmonary exercise testing. Depending on whether patients accepted or refused individualized exercise prescriptions based on cardio-pulmonary exercise testing (CPX) in Phase I cardiac rehabilitation, they were assigned to the individualized exercise prescriptions (IEP) group (*n* = 118) or the non-individualized exercise prescriptions (NIEP) group (*n* = 381). In the IEP group, the intensity of exercise was formulated base on each patient’s cardio-respiratory fitness (CRF) from CPX data [7]. In the NIEP group, the intensity of exercise was limited to Borg 11–13 by subjective sensation [7]. We compared the incidence of major cardiovascular events (MACE) between the two groups. IEP group were further divided into two subgroups based on prognosis status, namely the good prognosis (GP) group (*n* = 88) and the poor prognosis (PP) group (*n* = 30). By comparing the CRF between the two groups, we identify key CPX variables that may affect the prognosis of patients.

### Quantification of CRF

To accurately quantify CRF, we used CPX which is a widely accepted evaluation tool in both the United States (US) and Europe^.^[8, 9]. The measurement of ventilatory gas exchange was used for function-based prognostic stratification [9–11]. In the IEP group, oxygen consumption (VO_2_), carbon dioxide production (VCO_2_), minute ventilation (V_E_), partial pressure of end-tidal carbon dioxide (P_ET_CO_2_), and respiratory exchange ratio (RER) were measured in standard exercise testing using Cardio-respiratory instrumentation Medisoft (MS, made in Belgium, SN:130619–05-1470, Model: E100000011000001). The exercise tolerance was estimated from bicycle cycle ergometer work rate. The use of CPX during progressive exercise (10 watts per minute) is based on measurement of exercise gas exchange. The exercise test was terminated if any of the following occurred: abnormal hemodynamic or ECG exercise response or other reasons (i.e., lower extremity muscle fatigue, angina and dyspnoea).

### Clinical follow-up

Major adverse cardiac events (MACE) included cardio-genic death, re-hospitalization, heart failure, stroke, and atrial fibrillation. Follow-up data were collected through hospital records and telephone interviews which was conducted every 3 months after discharge until death or December 1, 2017, whichever came first. Mortality data for patients who were lost to telephone follow up were obtained from the population registry bureau. The average follow-up time was 2.5 years.

### Statistical analysis

For continuous variables, depending on whether a variable follows a normal distribution, the mean or median was reported and the t-test or nonparametric Wilcoxon’s rank sum test was applied for group comparison. The Chi-square tests were used for categorical variables. Multivariable logistic regression, in which we included age, cardiac function, test indices and variables showing a *p*-value< 0.05 in the univariate analysis, was used to identify independent risk factors for prognosis. The ROC curve was used to evaluate the predictive value of the model for MACE. All statistical analyses were done using SPSS 19 software (IBM Corp., Armonk, NY, USA).

## Results

### Incidence of major cardiovascular events

The patients’ clinical data are shown in Table 2. No significant difference in demographics was found between the IEP group and the NIEP group.

**Table 2:** Clinical data of the two study groups

	IEP group (*n* = 118)	NIEP group (*n* = 381)	*P*
Age, median (IQR)	57.0 (50.0, 62.3)	58.0 (51.0, 64.0)	0.363
Sex, male (%)	96 (81.4%)	291 (76.4%)	0.310
Extensive anterior wall MI (%)	27 (22.9%)	93 (24.4%)	0.830
Killip class ≥II (%)	17 (14.4%)	64 (18.6%)	0.640
Stenotic vessels ≥2 (%)	97 (82.2%)	337 (88.5%)	0.120
WBC (10^9^/L), median (IQR)	9.2 (7.7, 11.0)	9.5 (7.3, 12.3)	0.396
HGB (g/L), median (IQR)	148.0 (137.3, 158.0)	144.0 (133.0, 154.0)	0.082
Cr (umol/L), median (IQR)	75.5 (64.3, 84.8)	72.6 (61.5, 86.7)	0.306
AST (U/L), median (IQR)	60.5 (28.5, 132.4)	66.1 (29.5, 147.7)	0.413
ALT (U/L), median (IQR)	38.2 (23.2, 59.1)	37.2 (22.8, 54.0)	0.393
TC (mmol/L), median (IQR)	4.5 (3.9, 5.0)	4.5 (3.8, 5.1)	0.915
HDL-C (mmol/L), median (IQR)	1.0 (0.9, 1.2)	1.1 (0.9, 1.3)	0.054
LDL-C (mmol/L), median (IQR)	2.9 (2.4, 3.4)	2.8 (2.3, 3.3)	0.249
Fasting blood sugar (mmol/L), median (IQR)	6.1 (5.1, 7.3)	6.1 (5.1, 7.6)	0.526

*IEP group* Individualized exercise prescriptions group, *NIEP group* Non-individualized exercise prescriptions group, *extensive anterior wall MI* Extensive anterior wall myocardial infarction, *WBC* White blood cell, *HGB* Hemoglobin, *Cr* Creatinine, *AST* Glutamic pyruvic transaminase, *ALT* Glutamic pyruvic aminotransferase, *TC* Total cholesterol, *HDL-C* High density lipoprotein cholesterol, *LDL-C* Low density lipoprotein cholesterol, *IQR* Interquartile range

**Fig. 1:**
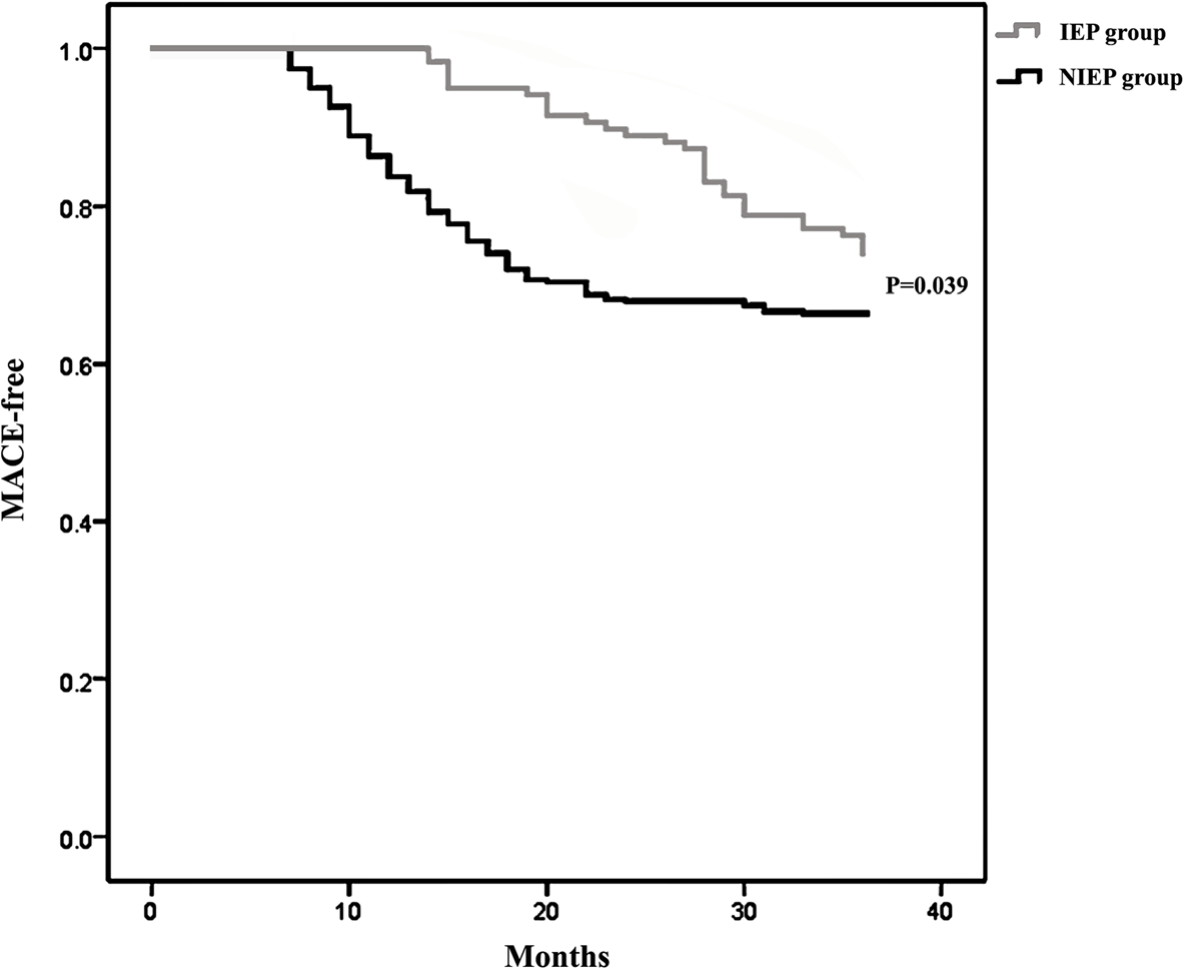
The Kaplan-Mayer curves of MACE-free survival. IEP group: individualized exercise prescriptions group; NIEP group: non-individualized exercise prescriptions group; MACE: major cardiac events

The results of adverse events are shown in Table 3. There was no significant difference in the incidence of cardio-genetic death (3 vs. 18, *P* = 0.442), re-hospitalization (27 vs. 109, *P* = 0.270), heart failure (3 vs. 13, *P* = 0.865), stroke (1 vs. 10, *P* = 0.429), or atrial fibrillation (0 vs. 2, *P* = 1.000) between the IEP and the NIEP groups. But the incidence of total MACE was significantly lower in the IEP group than in the NIEP group (34 vs. 152, *P* = 0.039; Fig. 1).

**Table 3:** Comparison of two groups of MACE (2 years)

	IEP group (*n* = 118)	NIEP group (*n* = 381)	*P*
Cardiogenic death, n (%)	3 (2.5%)	18 (4.7%)	0.442
Rehospitalization, n (%)	27 (22.9%)	109 (28.6%)	0.270
Heart failure, n (%)	3 (2.5%)	13 (3.4%)	0.865
Stroke, n (%)	1 (0.9%)	10 (2.6%)	0.429
Atrial fibrillation, n (%)	0 (0.0%)	2 (0.5%)	1.000
MACE, n (%)	34 (28.8%)	152 (39.9%)	0.039

*IEP group* Individualized exercise prescriptions group, *NIEP group* Non-individualized exercise prescriptions group, *MACE* Major cardiac events

### The key CPX variables affecting prognosis

The clinical data of the patients in the IEP group are summarized in Table 4. No significant difference was found between the GP subgroup and the PP subgroup.

**Table 4:** Clinical data of the patients in the PP and GP group

	PP group (*n* = 30)	GP group (*n* = 88)	*P*
Age (years)	57.9 ± 8.7	56.2 ± 9.8	0.379
Sex, male (%)	22 (73.3%)	75 (85.2%)	0.232
Extensive anterior wall MI(n)	9 (30.0%)	18 (20.5%)	0.410
Killip class ≥II (n)	6 (20.0%)	11 (12.5%)	0.478
Stenotic vessels ≥2 (n)	24 (80.0%)	73 (83.0%)	0.929
WBC(10^9^/L), median (IQR)	8.8 (7.5, 12.0)	9.4 (7.8, 10.9)	0.800
HGB(g/L)	139.9 ± 20.7	147.4 ± 16.1	0.080
Cr (umol/L), median (IQR)	77.1 (69.7, 85.5)	75.4 (63.3, 85.0)	0.636
AST(U/L), median (IQR)	58.3 (26.6, 156.1)	60.5 (28.8, 113.2)	0.858
ALT(U/L), median (IQR)	41.3 (22.4, 64.1)	37.5 (22.6, 58.4)	0.807
TC (mmol/L), median (IQR)	4.5 (4.1, 4.7)	4.5 (3.9, 5.1)	0.961
HDL-C (mmol/L), median (IQR)	1.1 (0.9, 1.2)	1.0 (0.9, 1.3)	0.683
LDL-C (mmol/L), median (IQR)	2.7 (2.4, 3.1)	3.0 (2.4, 3.5)	0.232
Fasting blood sugar (mmol/L), median (IQR)	6.0 (5.0, 7.3)	6.1 (5.2, 7.6)	0.663

*PP group* Poor prognosis group, *GP group* Good prognosis group, *Extensive anterior wall MI* Extensive anterior wall myocardial infarction, *WBC* White blood cell, *HGB* Hemoglobin, *Cr* Creatinine, *AST* Glutamic pyruvic transaminase, *ALT* Glutamic pyruvic aminotransferase, *TC* Total cholesterol, *HDL-C* High density lipoprotein cholesterol, *LDL-C* Low density lipoprotein cholesterol, *IQR* Interquartile range

The VO_2_ at VT, E-VCO_2_, △CO_2_, R-_PET_CO_2_, E-_PET_CO_2_ and △_PET_CO_2_ were significantly higher in the GP subgroup than in the PP subgroup (*P* = 0.006, *P* = 0.017, *P* = 0.018, *P* = 0.045, *P* = 0.005 and *P* = 0.022, respectively; Table 5). The VE/VCO2 slope was significantly lower in the GP subgroup than in the PP subgroup (*P* = 0.010). There were no statistically significant differences in other parameters.

**Table 5:** Comparison of cardiopulmonary exercise test results of patients with different prognosis

	PP group (*n* = 30)	GP group (*n* = 88)	*P*
VO_2_ at VT (ml/kg/min)	10.0 (8.8, 12.0)	12.0 (10.0, 14.0)	0.006
Ve/VCO_2_ slope	36.0 (32.3, 43.9)	33.0 (30.2, 38.1)	0.010
R-HR (bpm)	74.5 (63.0, 89.0)	73.5 (68.0, 81.8)	0.889
R-VCO_2_(L/min)	0.2 (0.2, 0.3)	0.2 (0.2, 0.3)	0.602
R-Ve(L/min)	12.2 ± 2.3	12.2 ± 2.1	0.862
E-HR (bpm)	90.0 (80.8, 111.5)	95.0 (86.3, 103.8)	0.899
E-VCO_2_(L/min)	0.6 ± 0.2	0.7 ± 0.2	0.017
E-Ve(L/min)	26.2 ± 6.1	27.2 ± 5.1	0.421
△Ve(L/min)	14.0 (11.1, 15.8)	14.4 (11.9, 18.6)	0.311
R-P_ET_CO_2_ (mm Hg)	29.0 (26.0, 31.3)	31.0 (28.3, 33.0)	0.045
E-P_ET_CO_2_ (mm Hg)	32.0 (29.0, 35.0)	35.0 (32.0, 37.0)	0.005
△PETCO2(mm Hg)	3.0 (2.0, 5.0)	4.0 (3.0, 6.0)	0.022
E-Ve/M (%)	26.0 (21.8, 28.3)	25.0 (22.3, 30.0)	0.843
△CO2(L/min)	0.4 ± 0.1	0.5 ± 0.2	0.018

*PP group* Poor prognosis group, *GP group* Good prognosis group, *VO*_*2*_ *at VT* Oxygen consumption per kilogram of weight per minute at anaerobic threshold, *Ve/VCO*_*2*_ *slope* Minute ventilation/ Carbon dioxide production slope, *R-HR* Rest heart rate, *R-VCO*_*2*_ Rest carbon dioxide production, *R-Ve* Rest minute ventilation, *E-HR* Exercise heart rate, *E-VCO*_*2*_ Exercise carbon dioxide production, *E-Ve* Exercise minute ventilation, △*Ve* Margin of minute ventilation, *R-P*_*ET*_*CO*_*2*_ Rest partial pressure of end-tidal carbon dioxide, *E-P*_*ET*_*CO*_*2*_ Exercise partial pressure of end-tidal carbon dioxide, △*P*_*ET*_*CO*_*2*_ Margin of partial pressure of end-tidal carbon dioxide, *E-Ve/M* Ratio of exercise minute ventilation to the maximum expected value, △*CO*_*2*_ Margin of Minute ventilation carbon dioxide production

**Fig. 2:**
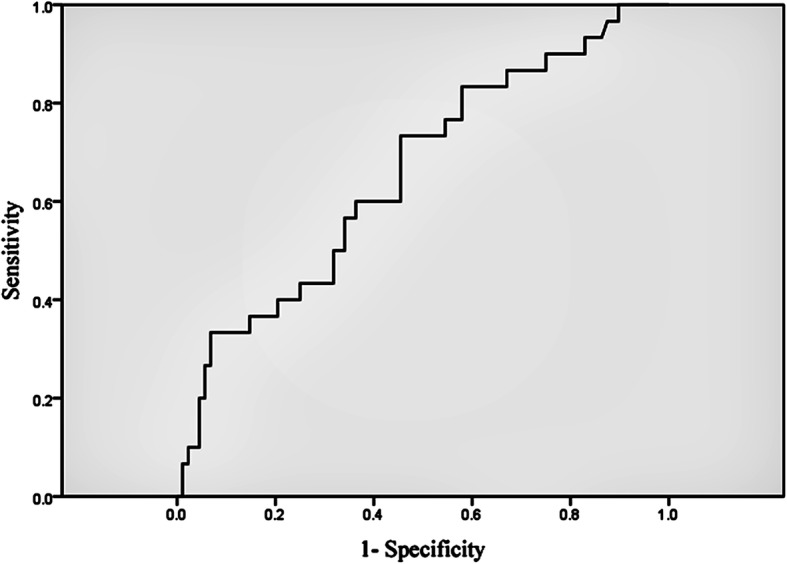
The ROC curve of Ve/VCO_2_ slope. Ve/VCO_2_ slope: Slope for minute ventilation/carbon dioxide production

**Fig. 3:**
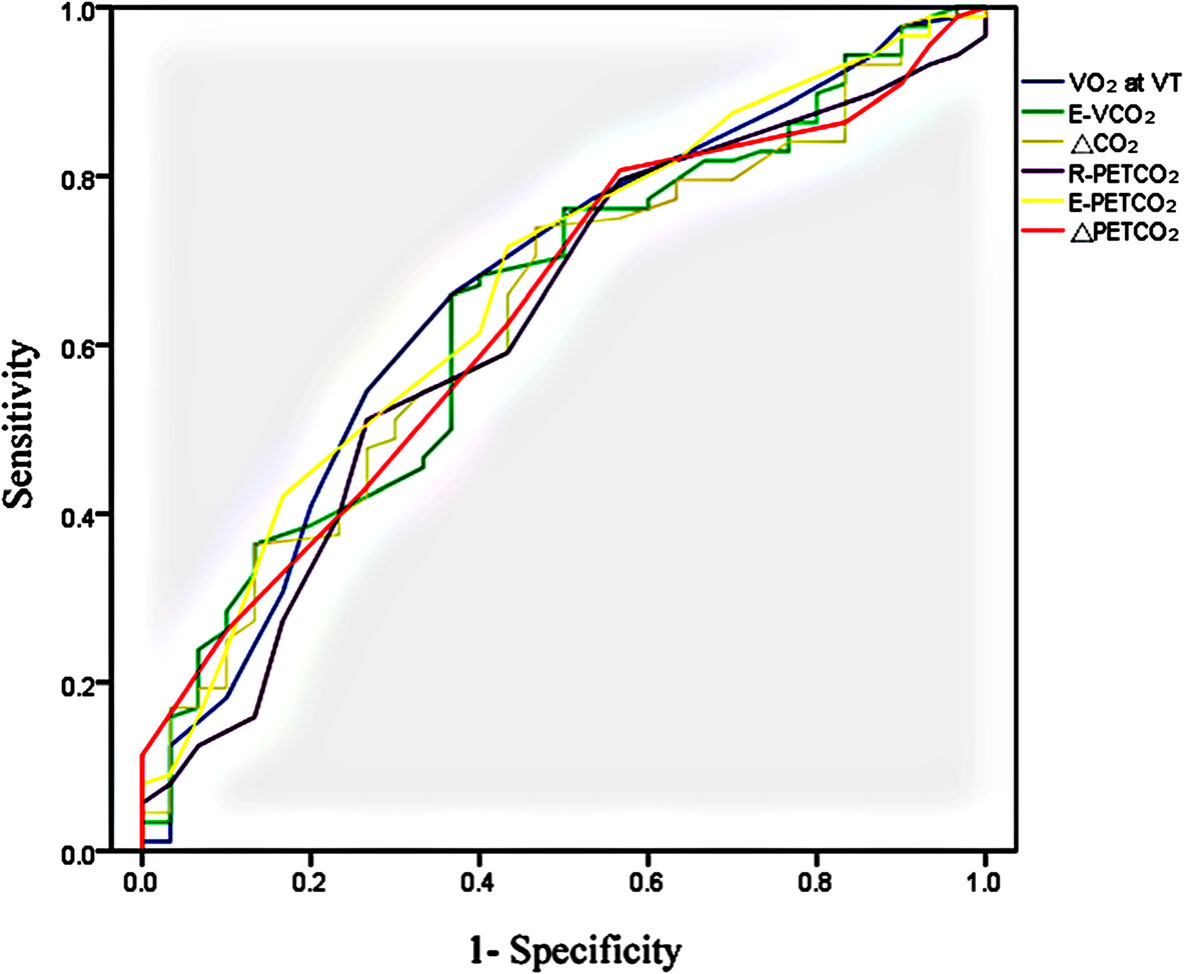
The ROC curve of VO_2_ at VT, E-VCO_2_, △CO_2_, R-PETCO_2_, E-PETCO_2_, and △PETCO_2_. VO_2_ at VT: Oxygen consumption per kilogram of weight per minute at anaerobic threshold; E-VCO_2_: Exercise carbon dioxide production; △CO_2_: Margin of Minute ventilation carbon dioxide production; R-P_ET_CO_2_: Rest partial pressure of end-tidal carbon dioxide; E-P_ET_CO_2_: Exercise partial pressure of end-tidal carbon dioxide; △P_ET_CO_2_: Margin of partial pressure of end-tidal carbon dioxide

**Fig. 4:**
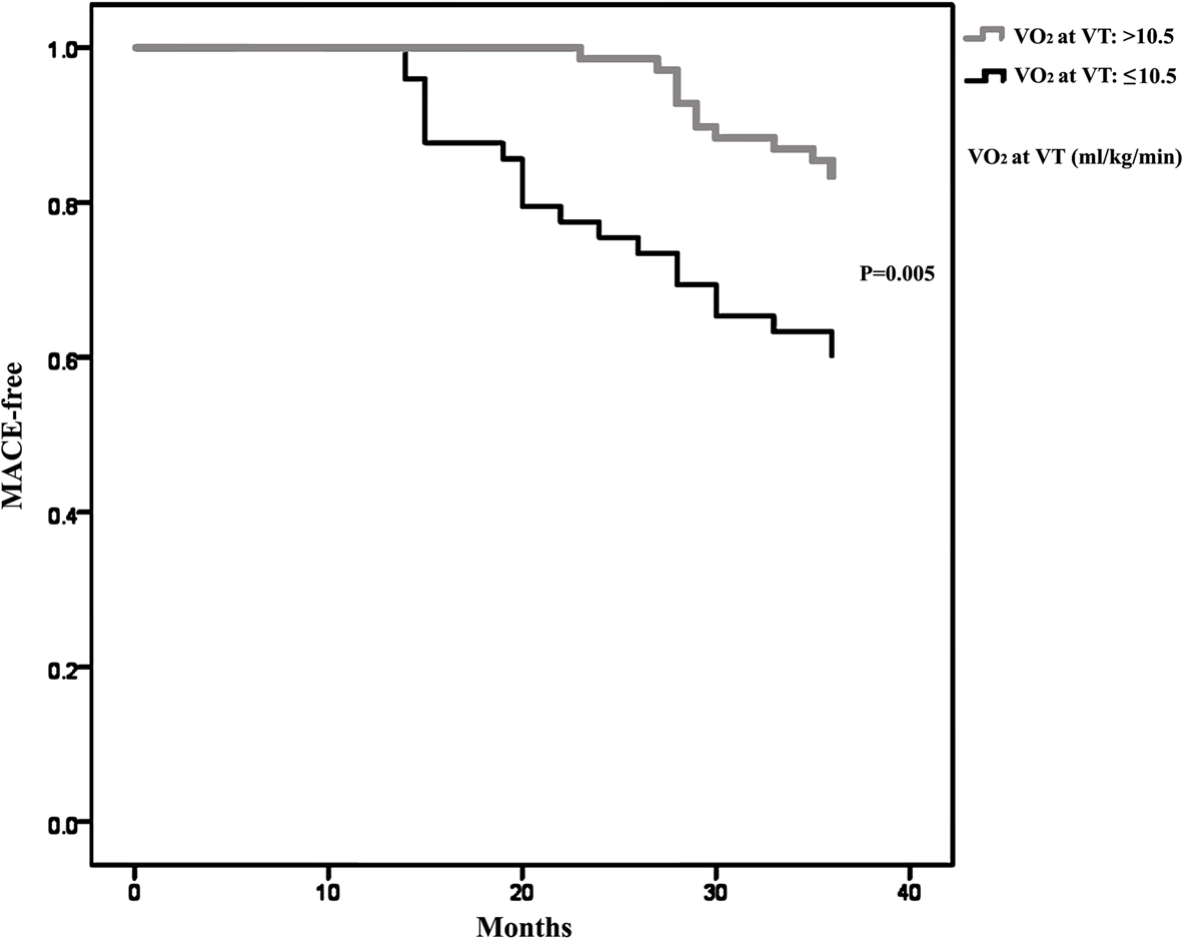
The Kaplan-Mayer curves of MACE-free survival. VO_2_ at VT: Oxygen consumption per kilogram of weight per minute at anaerobic threshold, MACE: major cardiac events

We found that VO_2_ at VT, V_E_/VCO_2_ slope, E-VCO_2_, △CO_2_, R-P_ET_CO_2_, E-PETCO_2_ and △PETCO_2_ to be predictive of adverse events (the areas under the curve being 0.666, 0.658, 0.646, 0.636, 0.623, 0.670 and 0.638, respectively), and the optimal cut-off point was 10.5 ml/kg/min, 33.4, 0.635 L/min, 0.345 L/min, 30.5 mmHg, 32.5 mmHg, and 2.5 mmHg, respectively (Figs. 2 and 3, Table 6). The VO_2_ at VT was an independent risk factor for cardiovascular disease prognosis (OR = 0.732, 95% CI: 0.541–0.988, *P* = 0.042; Table 7). The incidence of cardio-genetic death (0 vs. 3, *P* = 0.037), re-hospitalization (11 vs. 16, *P* = 0.033), and total MACE (13 vs. 21, *P* = 0.005) was significantly lower when the VO_2_ at VT was greater than 10.5 ml/kg/min (Table 8, Fig. 4).

**Table 6:** Optimal cut-off points and related diagnostic value by ROC analysis

	Cut-off point	Sensitivity	Specificity	AUC	*P*
VO_2_ at VT (ml/kg/min)	10.5	0.659	0.633	0.666	0.007
Ve/VCO_2_ slope	33.4	0.733	0.545	0.658	0.001
E-VCO_2_(L/min)	0.6	0.659	0.633	0.646	0.017
R-P_ET_CO_2_ (mm Hg)	30.5	0.511	0.733	0.623	0.046
E-P_ET_CO_2_ (mm Hg)	32.5	0.716	0.567	0.67	0.005
△P_ET_CO_2_(mm Hg)	2.5	0.807	0.433	0.638	0.024
△CO_2_(L/min)	0.3	0.739	0.533	0.636	0.027

*VO*_*2*_ *at VT* Oxygen consumption per kilogram of weight per minute at anaerobic threshold, *Ve/VCO*_*2*_ *slope* Minute ventilation/ Carbon dioxide production slope, *E-VCO*_*2*_ Exercise carbon dioxide production, *R-P*_*ET*_*CO*_*2*_ Rest partial pressure of end-tidal carbon dioxide, *E-P*_*ET*_*CO*_*2*_ Exercise partial pressure of end-tidal carbon dioxide, △*P*_*ET*_*CO*_*2*_ Margin of partial pressure of end-tidal carbon dioxide, △*CO*_*2*_ Margin of minute ventilation carbon dioxide production, *AUC* Area under the curve

**Table 7:** Results of logistic regression

	OR	95% CI	*P*
Age	1.013	0.952–1.077	0.688
Sex, male	2.277	0.302–17.153	0.425
Extensive anterior wall MI	2.300	0.656–8.068	0.193
Killip class ≥II	1.189	0.247–5.730	0.829
Stenotic vessels ≥2	0.605	0.148–2.471	0.484
WBC	0.993	0.813–1.214	0.945
HGB	1.001	0.958–1.046	0.964
Cr	1.000	0.983–1.017	0.988
AST	1.000	0.996–1.005	0.863
ALT	1.002	0.996–1.009	0.489
TC	1.433	0.736–2.787	0.290
HDL-C	0.803	0.385–1.674	0.558
LDL-C	0.487	0.212–1.118	0.090
Fasting blood sugar	0.910	0.702–1.179	0.476
VO_2_ at VT	0.732	0.541–0.988	0.042
Ve/VCO_2_ slope	0.903	0.744–1.096	0.302
R-HR	0.944	0.878–1.015	0.120
R-VCO_2_	1.8E+ 09	0.000–1.3E+ 22	0.158
R-Ve	0.650	0.298–1.417	0.279
E-HR	1.050	0.982–1.122	0.153
E-VCO_2_	0.000	0.000–1.9E+ 03	0.221
E-Ve	1.543	0.864–2.755	0.142
R-P_ET_CO_2_	0.955	0.634–1.438	0.825
E-P_ET_CO_2_	0.922	0.573–1.484	0.739
E-Ve/M	0.936	0.813–1.078	0.359

*Extensive anterior wall MI* Extensive anterior wall myocardial infarction, *WBC* White blood cell, *HGB* Hemoglobin, *Cr* Creatinine, *AST* Glutamic pyruvic transaminase, *ALT* Glutamic pyruvic aminotransferase, *TC* Total cholesterol, *HDL-C* High density lipoprotein cholesterol, *LDL-C* Low density lipoprotein cholesterol, *VO*_*2*_ *at VT* Oxygen consumption per kilogram of weight per minute at anaerobic threshold, *Ve/VCO*_*2*_ *slope* Minute ventilation/ carbon dioxide production slope, *R-HR* Rest heart rate, *R-VCO*_*2*_ Rest carbon dioxide production, *R-Ve* Rest minute ventilation, *E-HR* Exercise heart rate, *E-VCO*_*2*_ Exercise carbon dioxide production, *E-Ve* Exercise minute ventilation, *R-P*_*ET*_*CO*_*2*_ Rest partial pressure of end-tidal carbon dioxide, *E-P*_*ET*_*CO*_*2*_ Exercise partial pressure of end-tidal carbon dioxide, *E-Ve/M* Ratio of exercise minute ventilation to the maximum expected value, *OR* Odds ratio, *CI* Confidence interval

**Table 8:** Comparison of MACE by the cut-off points of VO_2_ at VT (2 years)

	> 10.5 ml/kg/min (*n* = 69)	≤ 10.5 ml/kg/min (*n* = 49)	*P*
Cardiogenic death, n (%)	0 (0.0%)	3 (6.1%)	0.037
Rehospitalization, n (%)	11 (15.9%)	16 (32.7%)	0.033
Heart failure, n (%)	1 (1.4%)	2 (4.1%)	0.371
Stroke, n (%)	1 (1.4%)	0 (0.0%)	0.397
Atrial fibrillation, n (%)	0 (0.0%)	0 (0.0%)	1.000
MACE, n (%)	13 (18.8%)	21 (42.9%)	0.005

*VO*_*2*_ *at VT* Oxygen consumption per kilogram of weight per minute at anaerobic threshold, *MACE* Major cardiac events

## Discussion

This study found that individualized exercise prescription of Phase I cardiac rehabilitation reduced the incidence of cardiovascular events in patients with STEMI after PCI. The key CPX variables, including VO_2_ at VT, V_E_/VCO_2_ slope, E-VCO_2_, △CO_2_, R-_PET_CO_2_, E- _PET_CO_2_ and △_PET_CO_2_ were predictive of these adverse events. Furthermore, the VO_2_ at VT was an independent risk factor for prognosis of cardiovascular disease.

Cardiac rehabilitation is a comprehensive treatment program that includes multiple components such as drug therapy, smoking cessation, exercise, psychology and nutrition. Phase I cardiac rehabilitation is in-hospital rehabilitation, which involves educating patients to recognize disease, prevent disease, and self-management. It can improve the prognosis of patients with unstable angina, acute myocardial infarction and heart failure [10, 11]. A large cohort study found that, in patients with coronary heart disease after revascularization, postoperative cardiac rehabilitations significantly reduced the mortality rate 1–5 years after operation [12]. Our study extends the prior finding by showing that individualized exercise prescription of Phase I cardiac rehabilitation can improve the total MACE of patients than traditional Phase I cardiac rehabilitation. It is somehow disappointing that we did not find significant differences between IEP and NIEP groups, regarding incidence of cardio-genetic death, re-hospitalization, heart failure, stroke, or atrial fibrillation. The reasons are as follows: first, cardiac rehabilitation include patient education, nutrition guidance, medication guidance, smoking cessation and psychological prescription, etc. except for exercise prescription. It shows that other Phase I cardiac rehabilitation parts also plays an important role in cardio-genetic death, re-hospitalization, heart failure, stroke, or atrial fibrillation [13]. Second, our study showed that cardio-genetic death, re-hospitalization, and total MACE decrease significantly when the VO_2_ at VT was greater than 10.5 ml/kg/min. Our results showed that pre-discharge cardiopulmonary fitness in Phase I cardiac rehabilitation could improve the long term prognosis of the STEMI patients. In addition, other study [14] also show that individualized exercise could not give any major advantage. These findings, taken together, suggest that individualized exercise prescription based on cardiopulmonary fitness was not only effective but also safe.

Exercise prescription guidance can be based on CPX or 6 min walk test (6MWT) [7]. This study adopted the CPX method, which was well recognized as the gold standard aerobic exercise assessment. The use of CRF has many utilizations in clinical, including measuring therapy progress and diagnosis. Although exercise tolerance normally came from the measurement of bicycle cycle ergometer work rate or treadmill, CPX is a more accurate tool to measure CRF. The measurement of CPX depends on the exchange of gases throughout exercise. CPX has got recognition not only it can give accurate measurement of patients with cardiovascular and pulmonary disease, but also it was easier to use because of technological progress, rapid response analyzers and computer-assisted data processing [7]. The 2016 EACPR/AHA scientific statement has indicated that peak VO_2_, VO_2_ at VT, and the minute ventilation/carbon dioxide production (V_E_/VCO_2_) relationship (V_E_/VCO_2_ slope) have prognostic significance [15].

Peak VO2 is defined as the highest O_2_ uptake obtained during exercise. Its response is resulting from central and peripheral functions, and widely indicates disease seriousness. Lots of studies have demonstrated that noninvasively determined peak cardiac output was regarded as a separate predictor of outcomes that improves the prognostic benefit of Peak VO_2_ [16–19]. Present evidence shows that if predicted peak VO_2_ value is below 50%, patients with heart failure (HF) have a poor prognosis in [20]. The prognostic value of VO_2_ at VT has been regarded to be a significant prognostic marker when evaluating pre-surgical risk using CPX [21, 22]. Importantly, an accurate and predictable recognition of VO_2_ at VT is not always possible which has been shown in patients with HF. If VO_2_ at VT is not predictable, the validity of the CPX should be accepted by providing that the subject attempt to reach an acceptable level (i.e., peak respiratory exchange ratio ≥ 1.00). The present study only uses this index to formulate exercise prescriptions. While the 2016 AHA guide pointed out that VO_2_ at VT is an independent risk factor for the prognosis of postoperative patients, it did not mention the implication of the index in STEMI patients.

In this study, we found that VO_2_ at VT is positively correlated with the prognosis of STEMI patients such that patients with VO_2_ at VT < 10.5 had poor cardiovascular prognosis. Therefore, VO_2_ at VT can be used as an important evaluation index of Phase I cardiac rehabilitation. It is safe and effective to formulate exercise prescription according to blood pressure, heart rate and watt under VO_2_ at VT. Large-scale randomized controlled trials are needed to confirm this.

V_E_/VCO_2_ slope represents matching of ventilation and perfusion within the pulmonary system, and broadly reflects disease severity as well as prognosis in several patient populations including HF, hypertrophic cardiomyopathy (HCM), pulmonary arterial hypertension (PAH)/secondary pulmonary hypertension (PH), chronic obstructive pulmonary disease (COPD), and interstitial lung Disease (ILD). A VE/V_CO2_ slope < 30 is considered normal while slight increase is possible with advanced age [7]. The index is usually used to assess the efficiency of ventilation, and involves in detecting high pulmonary pressures [23, 24]. Because pulmonary hypertension is often a consequence of left-sided valvular heart disease [25], the estimate of the V_E_/VCO_2_ slope may be particularly helpful. In many asymptomatic patients with severe aortic stenosis, it was found that elevated V_E_/VCO_2_ slope could be a significant predictor of decompensated HF or mortality [26]. Studies also showed that measures of ventilator efficiency, specifically the V_E_/VCO_2_ slope and P_ET_CO_2_, may be valuable in patients with HCM as these measures are implicated in increased pulmonary pressures [27, 28]. In this study, we found that V_E_/VCO_2_ slope was also a predictor of prognosis in STEMI patients after PCI, with a cut-off point value of 33.4, suggesting that patients with VE/VCO_2_ slope over 33.4 tended to have poorer prognosis. Impaired cardiac output leads to decreased aerobic metabolism, increased anaerobic metabolism, and decreased VCO_2_ emissions, which may result in increased VE/VCO_2_ slope.

In all the CPX variables in patients with systolic HF, peak VO_2_ and the V_E_/VCO_2_ slope have been shown stable separate prognostic significance. While V_E_/VCO_2_ slope is a stronger predictive marker in the univariate model compared with peak VO_2_, there is strong evidence that indicates that a multivariate approach may improve prognostic accuracy [7]. With current healthy management strategies, a VE/VCO_2_ slope ≥ 45, and a peak VO_2_/ kg/ min < 10.0 ml are indicative of poorer prognosis over a 4-year period following CPX [27]. In this study, likely because all the patients had acute STEMI, and most of them did not have respiratory dysfunction, the cut-off point of V_E_/VCO_2_ slope differed, but the cut-off point of VO_2_/kg/min was the same as the study of Arena, R. et al. [27].

As V_E_/VCO_2_ slope, P_ET_CO_2_ widely reflects disease severity in lots of patient with HF, HCM, PAH/secondary PH, COPD, and ILD. Both exercise oscillatory ventilation and P_ET_CO_2_ during rest and exercise have been shown to be of prognostic value in patients with systolic HF [29, 30]. Abnormalities in the V_E_/VCO_2_ slope and P_ET_CO_2_ have been thought to be pulmonary vasculopathy. We found that P_ET_CO_2_ was also a predictor of the prognosis of patients with STEMI after PCI, such that increased P_ET_CO_2_ was associated with a better prognosis. The difference in the heart function in the quiet/movement and P_ET_CO_2_ might be due to difference in infarct area of the patients.

## Conclusion

This study found that individualized exercise prescription of Phase I cardiac rehabilitation reduced the incidence of cardiovascular events in patients with STEMI after PCI, and long term prognosis of patients based on their pre-discharge cardiopulmonary fitness. The key CPX variables, including VO_2_ at VT, V_E_/VCO_2_ slope, E-VCO_2_, △CO_2_, R-P_ET_CO_2_, E-P_ET_CO_2_ and △P_ET_CO_2_, are predictive of MACE. VO_2_ at VT was an independent risk factor for cardiovascular disease prognosis and could be used as an important evaluating indicator for Phase I cardiac rehabilitation. Future studies with larger sample sizes are warranted to validate these findings.

## Data Availability

The data used to support the findings of this study are available from the corresponding author upon request.

## Abbreviations

△CO_2_: Margin of minute ventilation carbon dioxide production
△P_ET_CO_2_: Margin of partial pressure of end-tidal carbon dioxide
6MWT: 6 min walk test
AHA: American Heart Association
ALT: Glutamic pyruvic aminotransferase
AST: Glutamic pyruvic transaminase
COPD: Chronic obstructive pulmonary disease
CPX: Cardio-pulmonary exercise testing
CR: Cardiac rehabilitation
Cr: Creatinine
CRF: Cardio-respiratory fitness
E-VCO_2_: Exercise carbon dioxide production
GP: Good prognosis
HCM: Hypertrophic cardiomyopathy
HDL-C: High-density lipoprotein cholesterol
HF: Heart failure
HGB: Hemoglobin
IEP: Individualized exercise prescriptions
ILD: Interstitial lung disease
LDL-C: Low-density lipoprotein cholesterol
MACE: Major adverse cardiac events
NIEP: Non-individualized exercise prescriptions
PAH: Pulmonary arterial hypertension
PCI: Percutaneous coronary intervention
P_ET_CO_2_: Exercise partial pressure of end-tidal carbon dioxide
P_ET_CO_2_: Partial pressure of end-tidal carbon dioxide
P_ET_CO_2_: Rest partial pressure of end-tidal carbon dioxide
PH: Pulmonary hypertension
PP: Poor prognosis
RER: Respiratory exchange ratio
STEMI: ST-segment elevation myocardial infarction
TC: Total cholesterol
VCO_2_: Carbon dioxide production
V_E_: Minute ventilation
V_E_/VCO_2_ slope: Slope for minute ventilation/carbon dioxide production
VO_2_ at VT: Oxygen consumption per kilogram of weight per minute at anaerobic threshold
VO_2_: Oxygen consumption
VT: Ventilation threshold
WBC: White blood cell
